# Prostaglandin E_2_ from *Candida albicans* Stimulates the Growth of *Staphylococcus aureus* in Mixed Biofilms

**DOI:** 10.1371/journal.pone.0135404

**Published:** 2015-08-11

**Authors:** Jan Krause, Gernot Geginat, Ina Tammer

**Affiliations:** Institute of Medical Microbiology, Hospital Control and Prevention, Otto-von-Guericke University, Magdeburg, Germany; National University of Singapore, SINGAPORE

## Abstract

**Background:**

Previous studies showed that *Staphylococcus aureus* and *Candida albicans* interact synergistically in dual species biofilms resulting in enhanced mortality in animal models.

**Methodology/Principal Findings:**

The aim of the current study was to test possible candidate molecules which might mediate this synergistic interaction in an *in vitro* model of mixed biofilms, such as farnesol, tyrosol and prostaglandin (PG) E_2_. In mono-microbial and dual biofilms of *C*.*albicans* wild type strains PGE_2_ levels between 25 and 250 pg/mL were measured. Similar concentrations of purified PGE_2_ significantly enhanced *S*.*aureus* biofilm formation in a mode comparable to that observed in dual species biofilms. Supernatants of the null mutant deficient in PGE_2_ production did not stimulate the proliferation of *S*.*aureus* and the addition of the cyclooxygenase inhibitor indomethacin blocked the *S*.*aureus* biofilm formation in a dose-dependent manner. Additionally, *S*. *aureus* biofilm formation was boosted by low and inhibited by high farnesol concentrations. Supernatants of the farnesol-deficient *C*. *albicans* ATCC10231 strain significantly enhanced the biofilm formation of *S*. *aureus* but at a lower level than the farnesol producer SC5314. However, *C*. *albicans* ATCC10231 also produced PGE_2_ but amounts were significantly lower compared to SC5314.

**Conclusion/Significance:**

In conclision, we identified *C*. *albicans* PGE_2_ as a key molecule stimulating the growth and biofilm formation of *S*. *aureus* in dual *S*. *aureus/C*. *albicans* biofilms, although *C*. *albicans* derived farnesol, but not tyrosol, may also contribute to this effect but to a lesser extent.

## Introduction

The first study suggesting a specific interaction between *Staphylococcus* (*S*.) *aureus* and *Candida (C*.*) albicans* was published in 1976 [1]. Since then, a number of studies corroborated this result and showed a synergistic interaction of *S*. *aureus* and *C*. *albicans* with enhanced mortality in animal models [2–4].

*In vitro* studies of complex microbial communities show that intra-species and inter-species interactions are mediated via small molecules released into the extracellular environment, i.e. quorum sensing molecules, extracellular virulence factors, or secondary metabolites [5–7].

*Candida albicans* regulates virulence traits through the production of at least two quorum-sensing (QS) signal molecules, *E*,*E*-farnesol and tyrosol, both affecting dimorphism and biofilm formation in *C*. *albicans* [8–10]. Furthermore, *C*. *albicans* farnesol down-regulates the production of pyocyanin in *Pseudomonas (P*.*) aeruginosa* [11] and inhibits biofilm formation and lipase activity in *S*. *aureus* [12,13].

*Candida albicans* is a commensal microorganism in healthy individuals but is capable of causing disseminated or chronic infections when the host mucosal barrier is breached and the immune response is inadequate. *In vivo*, inflammation is mediated by the production of eicosanoids including prostaglandins and leukotrienes. Prostaglandin E_2_ (PGE_2_), an oxygenated metabolite of arachidonic acid, is known to regulate the activation, maturation, cytokine release and migration of the mammalian cells, particularly those involved in innate immunity [14–18]. Interestingly, *C*. *albicans* produces authentic PGE_2_ from external arachidonic acid [19], which is upregulated during biofilm formation suggesting that PGE_2_ may represent a significant virulence factor in biofilm-associated infections.

In order to evaluate the possible effect of PGE_2_ on bacterial biofilms, we established a *S*. *aureus/C*. *albicans* dual species biofilm model. Using this model we were able to replace the stimulatory activity of *C*. *albicans* on *S*. *aureus* by synthetic purified PGE_2_, suggesting that this metabolite plays an important role in the interaction of *S*. *aureus* and *C*. *albicans* in biofilms.

## Material and Methods

### *Staphylococcus aureus* and *C*. *albicans* strains

The following strains were used in this study: *Staphylococcus (S*.*) aureus* 31883 small colony variant (SCV) strain isolated from a sputum sample of a patient suffering from cystic fibrosis; *S*. *aureus* 19552 clinical isolate derived from the throat of a cystic fibrosis patient (non-SCV phenotype); *Candida (C*.*) albicans* 31883 isolated from a sputum sample of a patient suffering from cystic fibrosis; *C*. *albicans* ATCC10231 wild type (wt) strain (laboratory strain; farnesol-deficient); *C*. *albicans* SC5314 wt strain (laboratory strain; farnesol producer, [20]); *C*. *albicans* M35 (prototrophic reference strain; same as CAF2-1, but *ura3-/URA3* [21]); *C*. *albicans* M134 (prototrophic reference strain; same as CAI4, but *ura3-/- rps1*::*URA3/RPS*; [22]; further *ura3-/-*); *C*. *albicans* M1096 (prototrophic; same as CAI4, but *ura3-/- fet31-/-*::*URA3*; [23]; further *ura3-/-fet31-/-*).

Clinical isolates were cultured from routine diagnostic samples which were sent to the Institute of Medical Microbiology. The samples were processed in the diagnostic laboratory under the special guidance of the author as described below. After homogenization with sterile saline (1:1) the sputum samples were plated on various agar plates and incubated at 37°C and at 30°C for two days, respectively, and at 25°C for further three days. The throat swabs were processed in a similar manner without homogenization. After visible growth the colonies were identified by the use of mass spectrometry (Vitek2 MS; Biomerieux, France) according to the instructions of the manufacturer. All strains identified from patients with cystic fibrosis were routinely stored at -70°C using the Microbank system as recommended by the manufacturer (Bestbion; Colonia, Germany).

For the use in this study, clinical strains were cultured from the Microbank system on Columbia agar plates containing 5% sheep blood (Beckton Dickinson) and incubated over night at 37°C. Subcultures on blood agar plates were incubated for further 24h under the same conditions. From these subcultures glycerin stocks (99.9%; Calbiochem, Heidelberg, Germany) were prepared and stored at -70°C until further use.

### Dual *S*. *aureus/C*. *albicans* biofilms

Three to five *S*. *aureus* colonies grown from glycerin stocks were inoculated in 5mL trypcase soy broth (TSB-T; Biomerieux, Nürtingen, Germany) and incubated over night at 37°C with agitation of 180 rpm. *C*. *albicans* cells from glycerin stocks were propagated overnight in 100mL Yeast peptone dextrose (YPD) medium (Sigma, Hannover; Germany) at 30°C on an orbital shaker at 180 rpm. Under these conditions all strains grow in the budding yeast phase. *Candida* cells were harvested by centrifugation, washed twice in sterile phosphate buffered saline (1xPBS; Sigma), re-suspended and adjusted to a cellular density equivalent to 3x10^6^ cells/mL. *S*. *aureus* cells were adjusted to an optical density of 0.02 in RPMI1640 using a spectrophotometer (Eppendorf, Hamburg, Germany) which is equivalent to approximately 3x10^6^ cells/mL [24]. One mL of each microbial suspension was added to each well of a 12-well plate (Greiner, Frickenhausen, Germany). Biofilms were incubated in RPMI1640 buffered with HEPES and supplemented with L-glutamine and 10% heat-inactivated fetal bovine serum (FBS; Thermo Fisher Scientific, Schwerte, Germany) for the appropriate time at 37°C with 75 rpm of agitation in humid atmosphere. Medium was changed daily. Under these conditions *S*. *aureus* and *C*. *albicans* formed stable and reproducible mono-microbial and mixed biofilms which was tested using one-way ANOVA (analysis of variance) as described elsewhere [25,26]. After the appropriate time of incubation supernatants were removed. Biofilms were scraped from the bottom of the wells and homogenized with Sputasol liquid (50μg/mL; Thermo Fisher) as described by Efthimiadis et al. [27]. Cells were collected by centrifugation (1100xg for 5 min) and washed twice in sterile PBS. The inoculum was confirmed by quantitative culture (serial diluted 10-fold) on mannitol salt agar (MSA2 agar; Biomerieux) after 24h of incubation at 37°C for *S*. *aureus* or on Chromagar CANDIDA (BD) after 48h at 30°C for *C*. *albicans*.

### Crystal violet staining

Mono-microbial and mixed biofilms were stained with crystal violet (CV) solution as described by Peeters et al. [28]. Briefly, biofilms cultured in 96-well plates were fixed with methanol. Then, 0.2% crystal violet solution was added to each well and incubated for 20 min at room temperature. Excess CV was removed by washing under running tap water and bounded CV was released by 33% acetic acid (Sigma). Absorbance was measured at 570 nm.

### Determination of the prostaglandin concentration

Prostaglandin E_2_ (PGE_2_) production was measured from supernatants of different mono-microbial and dual biofilms using a monoclonal PGE_2_ enzyme-linked immunosorbent assay (ELISA; Cayman Chemicals, Ann Arbor, USA) according to the instructions of the manufacturer. Supernatants were collected from the biofilms after the appropriate time point, centrifuged at 8000xg, filtered twice by using a 0.22 μm syringe filter (Roth, Karlsruhe, Germany) and stored at -80°C. To confirm, that the supernatants yielded no living cells of *S*. *aureus* and *C*. *albicans*, 100 μl of the supernatants were plated onto blood agar plates and incubated for 48h at 37°C and for further 48h at 30°C. All supernatants processed by this method were cell-free. In serum, PGE_2_ is rapidly degraded into unstable intermediates [19], i.e. 15-keto-13, 14-dihydro-PGE_2_, which were determined with a PGE_2_ metabolite kit (Cayman Chemicals). The PGE_2_ metabolite kit converts the unstable intermediates into stable measurable derivative serving as marker for the PGE_2_ production. Background levels of PGE_2_ detected in RPMI 1640 with and without FBS were subtracted from experimental samples.

### Purified substances

Purified tyrosol, farnesol and prostaglandin E_2_ (PGE_2_) were purchased from Sigma-Aldrich. Stock solutions of tyrosol (2-4-hydroxyphenylethanol) and farnesol were dissolved in DMSO. PGE_2_ stock solution (1mg/mL) was prepared in ethanol and further diluted in 1xPBS. Established 24h old *S*. *aureus* biofilms were incubated overnight in the presence of the appropriate drugs, biofilms were harvested and the numbers of colony forming units (cfu`s) were determined as described above. Control experiments revealed that DMSO and ethanol did not affect to the growth of *S*. *aureus* and *C*. *albicans*.

### Inhibitors

The cyclooxygenase inhibitor indomethacin (Sigma) was dissolved in dimethyl sulfoxide (stock solution 1mM) and further diluted in PBS prior to addition to mixed biofilms. Dual biofilms without supplements or with DMSO alone were used as control. DMSO did not influence the growth of the biofilms.

### Statistical analysis

Data represent the mean plus the standard deviation of two independent experiments with three intra-assay replicates.

Statistical significance was determined using Student’s t-test with SigmaStat statistical software (version 2.0). *P*-values ≤ 0.05 were considered statistically significant.

### Ethics statement

According to the written decision of the clinical research ethics committee of the Otto-von-Guericke University of Magdeburg the current study did not require approval by the local ethics committee because no human material or data attributable to individual patients were used.

## Results

### Stimulation of *S*. *aureus* by *C*. *albicans* in mixed biofilms

**Fig 1:**
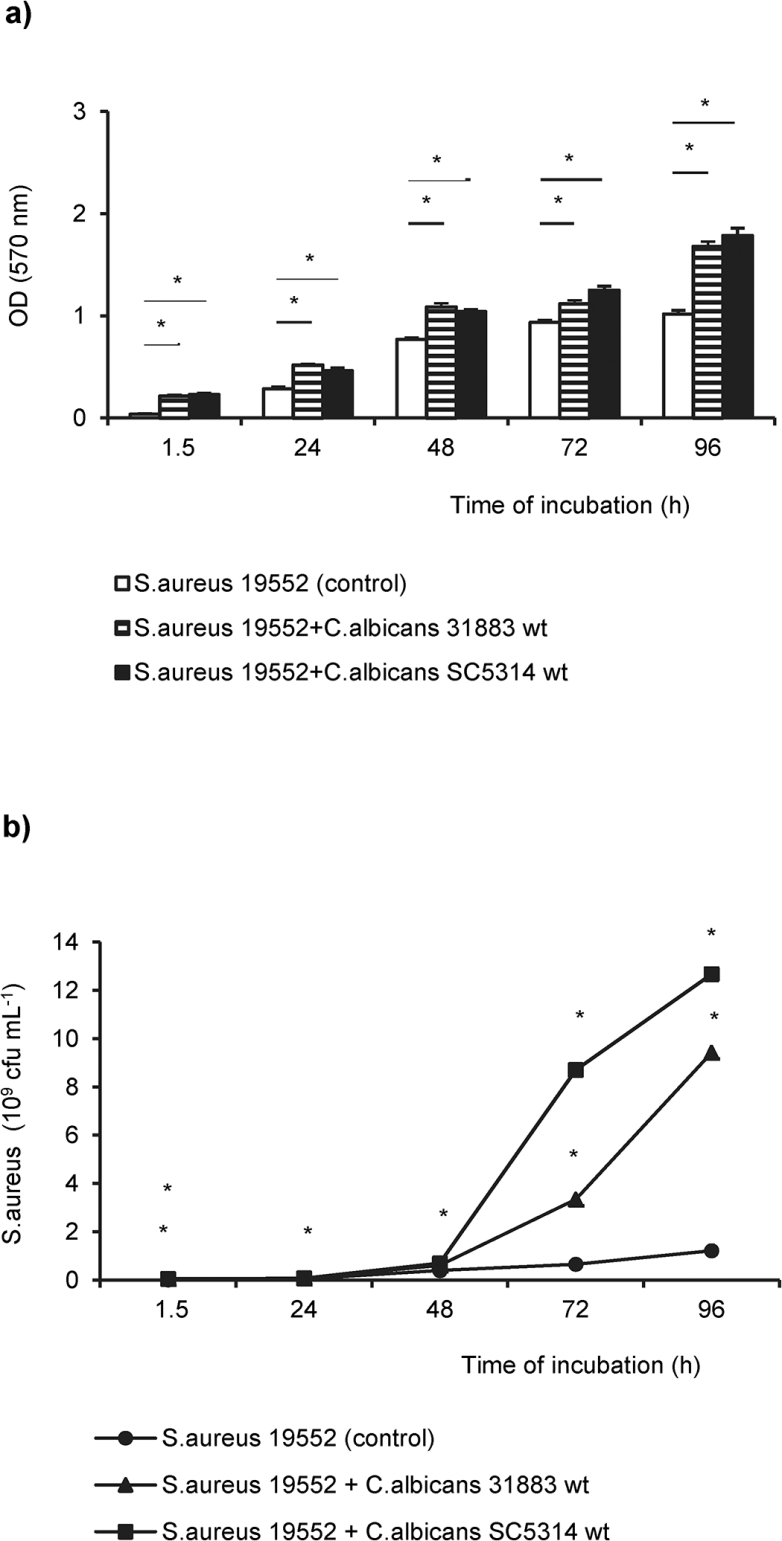
Growth kinetics of *S*.*aureus* in mono-microbial and in dual *S*. *aureus/C*. *albicans* biofilms. (**a**) Biofilm thickness of *S*. *aureus* 19552 single (open bars) and mixed biofilms with *C*. *albicans* 31883 wt (clinical strain; striped) or *C*. *albicans* SC5314 wt (laboratory strain; solid bars) at various time points determined by staining with crystal violet. Total biofilm thickness is expressed as mean of the optical density (OD) measured at a wave length of 570 nm. Shown are the means and standard deviations of two independent experiments with 24 replicates each. (**b**) Quantification of *S*. *aureus* 19552 in mono-microbial (circle) and in dual species biofilms together with *C*. *albicans* clinical isolate 31883 (triangle) or *C*. *albicans* SC5314 laboratory strain (square). Shown are the mean cfu`s of *S*. *aureus* derived from two representative experiments with three intra-assay replicates. Asterisks indicate significant (*p*<0.001) differences between mono-microbial and dual species biofilms.

Initial mixed biofilm experiments with clinical strains of *S*. *aureus* and *C*. *albicans* showed that biofilm thickness of mixed biofilms was significantly increased compared with *S*. *aureus* mono-microbial biofilms (Fig 1A).

Mixed biofilms yielded a significantly higher number of *S*. *aureus* colony forming units (cfu`s) compared to mono-microbial *S*. *aureus* biofilms with a dramatically increase after 72h of co-culture (Fig 1B).

**Fig 2:**
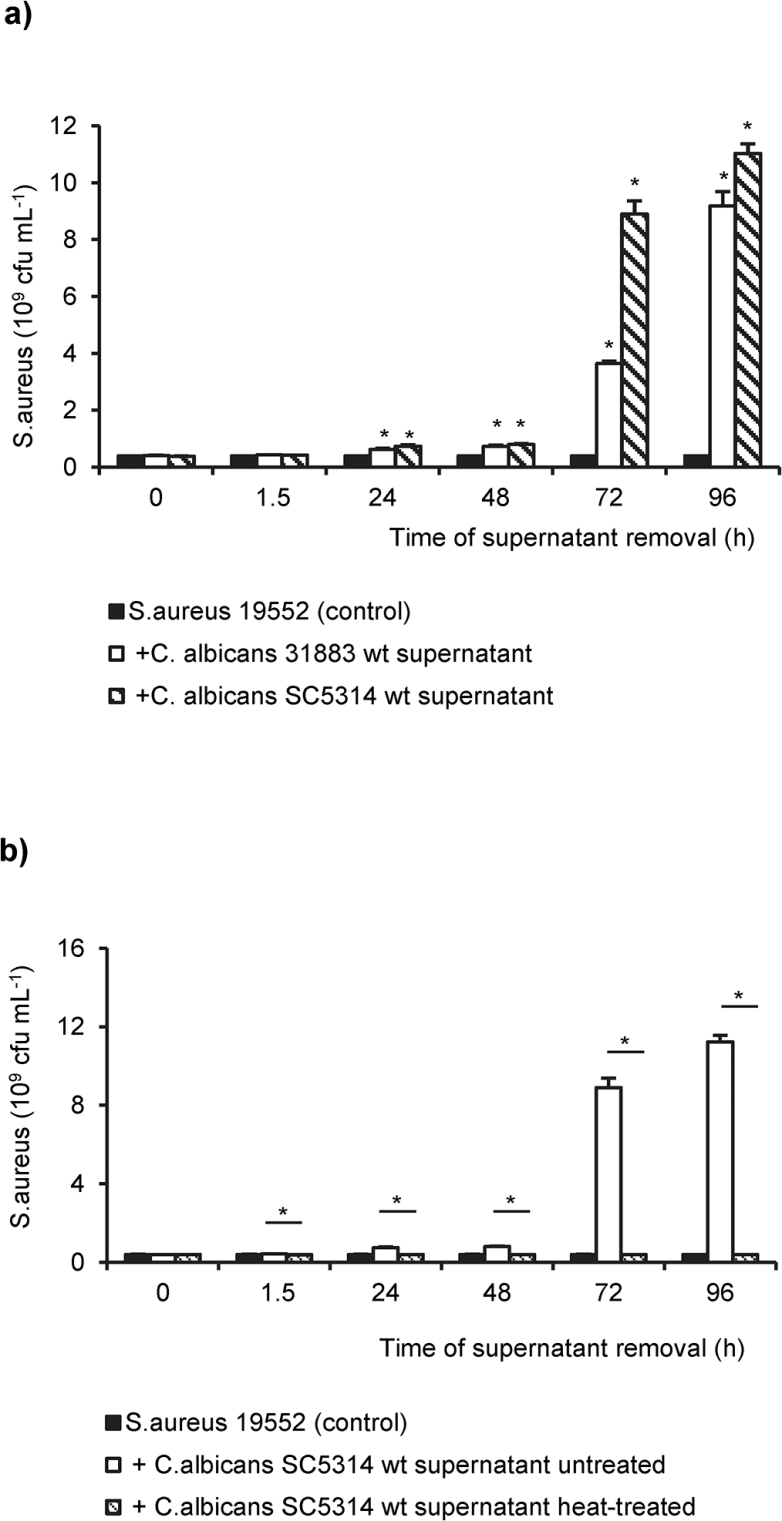
Growth-promoting properties of cell-free *C*. *albicans* supernatants. (**a**) Supernatants removed from biofilms of two *C*. *albicans* wild type strains SC5314 and 31883, respectively, after the indicated time of incubation were added to 24h old biofilms of *S*. *aureus* 19552. (**b**) Heat-treated (striped) and untreated supernatants (open bars) were collected as described above and added to 24h-old *S*. *aureus* biofilms. *S*. *aureus* grown as mono-microbial biofilm was used as control (solid bars). Shown are the mean cfu and the standard deviations of two independent experiments each with three replicates. Asterisks indicate statistically significant differences (*p*<0.001).

In order to investigate, if the growth-stimulating effect of *C*. *albicans* depends on the direct contact of bacteria to the hyphal network, 24h old *S*. *aureus* biofilms were incubated with cell-free supernatants from *C*. *albicans* 31883 and SC5314 diluted 1:1 with fresh medium (Fig 2A). The number of cfu`s of *S*. *aureus* increased 10-fold when supernatants derived from 72h or 96h old *C*. *albicans* biofilms were added. The strongest increase was observed with supernatants from *C*. *albicans* SC5314. Remarkably, the stimulatory activity of supernatants was heat-labile. Incubation of *S*. *aureus* with heat-treated supernatants from *C*. *albicans* mono-microbial biofilms did not promote the propagation of *S*. *aureus* (Fig 2B).

### Subinhibitory concentrations of farnesol promote biofilm formation of *S*. *aureus*

In order to further define the growth-promoting activity of *C*. *albicans* culture supernatants the effect of a number of molecules secreted by *C*. *albicans* such as farnesol, tyrosol and prostaglandin E_2_ (PGE_2_), were tested.

**Fig 3:**
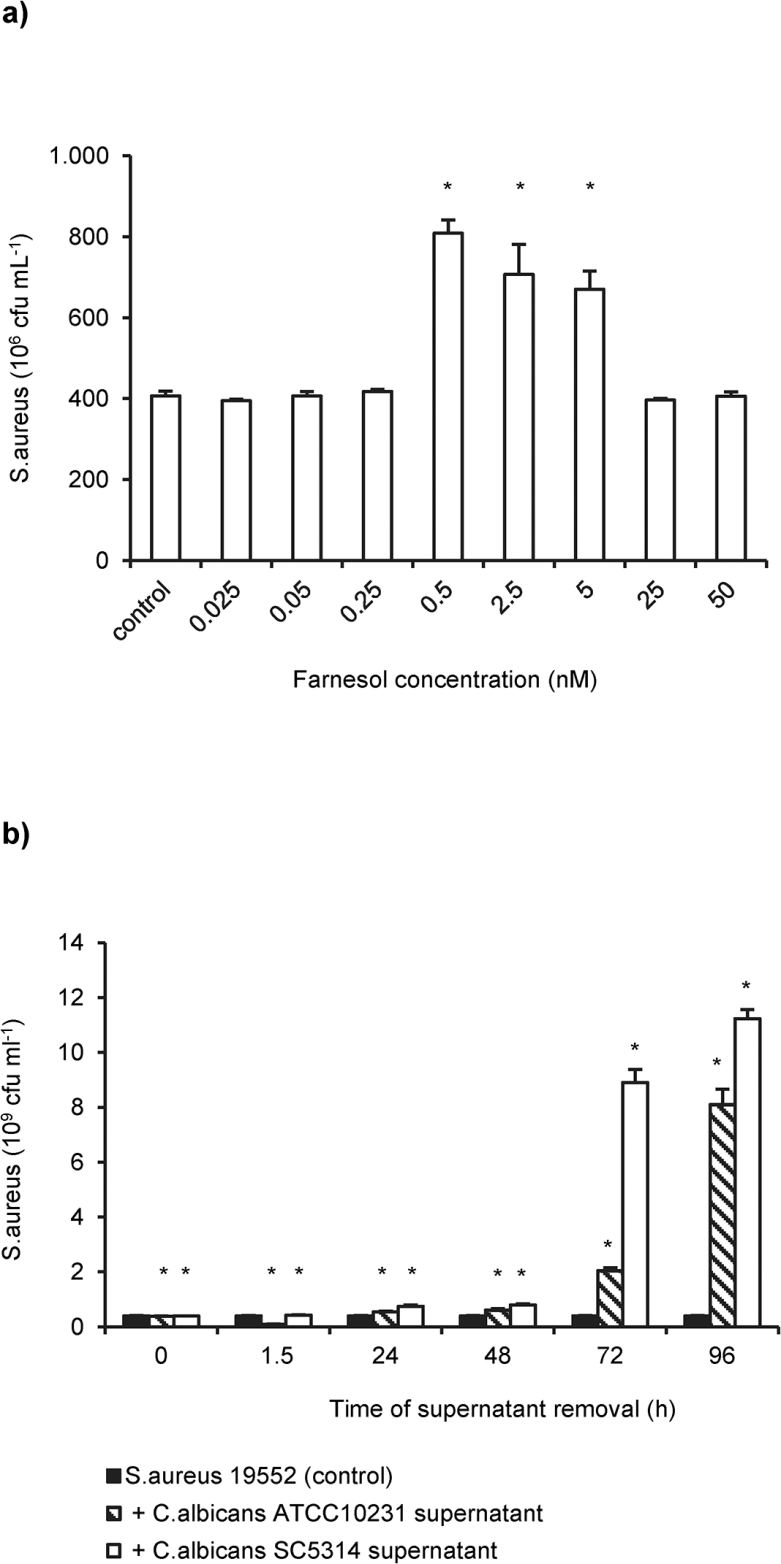
Effect of farnesol on the growth of *S*. *aureus* 19552 in mono-microbial biofilms. (**a**) Pre-cultered 24h old *S*. *aureus* biofilms were supplemented with different concentrations of purified farnesol and the number of cfu`s was determined. *S*. *aureus* biofilms incubated in farnesol-free medium were used as control. (**b**) Growth kinetics of *S*. *aureus* in dual biofilms with the farnesol-deficient *C*. *albicans* strain ATCC10231 (striped) and the farnesol producer *C*. *albicans* SC5314 (open bars). Colony forming units (cfu`s) of *S*. *aureus* derived from single species biofilms were function as control (solid bars). Shown are the mean cfu`s and the standard deviations of two representative experiments with three replicates each. Asterisks indicate significant (p<0.05) differences compared to controls.

Synthetic farnesol at concentrations between 5 and 0.5 nM (Fig 3A), but not purified tyrosol, stimulated the growth of *S*. *aureus* in biofilms, whereas concentrations ≥ 0.5 μM significantly reduced the growth of *S*. *aureus* (S1 Fig).

The application of supernatants from cultures of both *C*. *albicans* SC5314 (farnesol producer) and ATCC10231 (farnesol nonproducer) significantly enhanced the growth rates of *S*. *aureus* (Fig 3B). Interestingly, the farnesol-producing strain exhibited a stronger impact to the growth and biofilm formation of *S*. *aureus* than the farnesol-negative isolate.

### Purified PGE_2_ stimulates growth of *S*. *aureus* in biofilms

**Fig 4:**
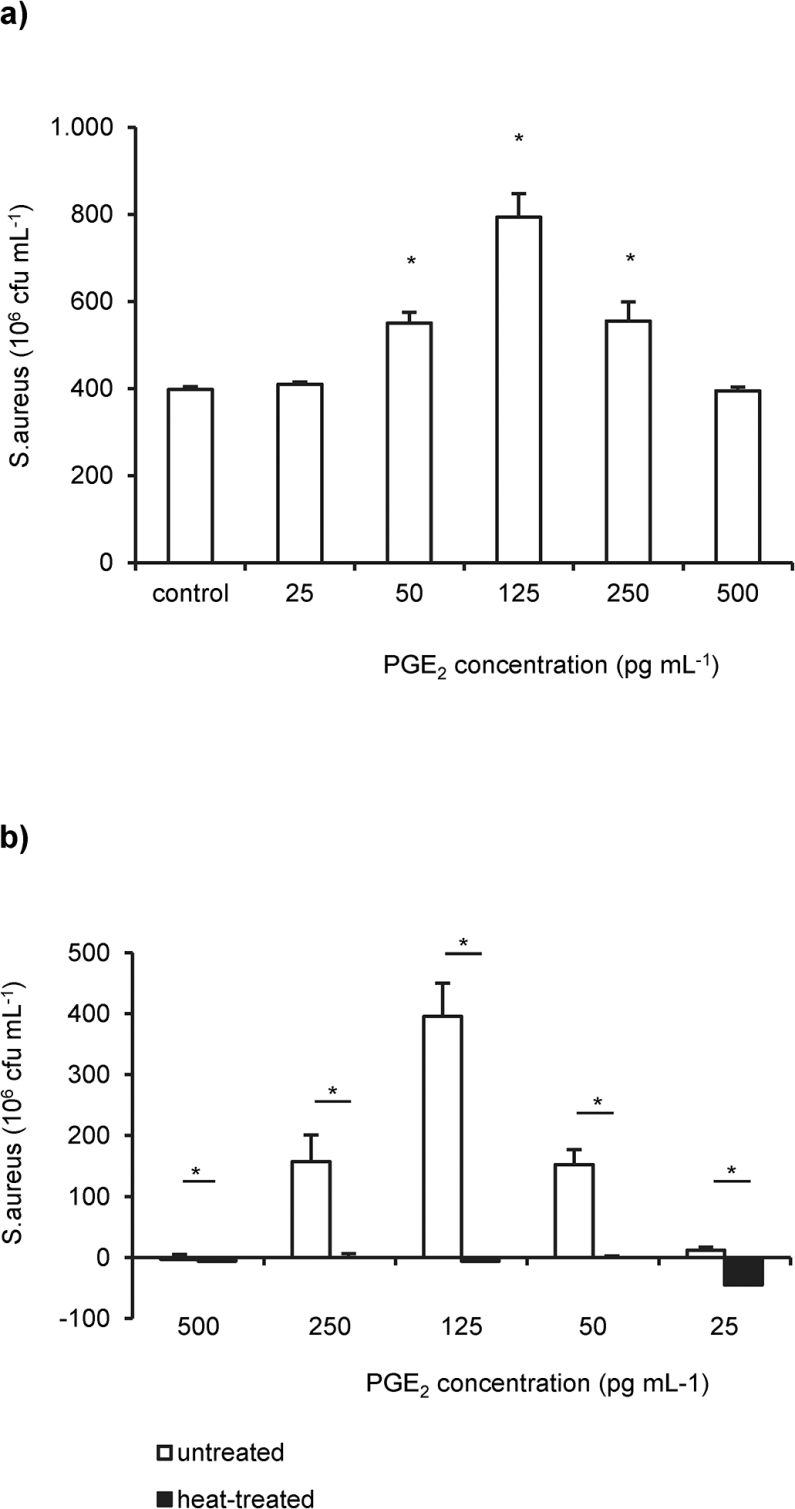
PGE_2_ stimulates the growth of *S*. *aureus* biofilms. (**a**) *S*. *aureus* 19552 was grown in single species biofilms in the presence of various concentrations of purified PGE_2._ The mean number of colony forming units (cfu`s) from untreated *S*. *aureus* biofilms were used as control. Asteriks indicate statistically significant (p< 0.05) differences between PGE_2_-treated and untreated biofilms. (**b**) Cultures of *S*. *aureus* 19552 biofilms were supplemented with heat-treated (solid bars) and untreated (open bars) PGE_2_ supplemented medium. Shown are the mean number of *S*. *aureus* cfu`s normalized to the non-supplemented control as well as the standard deviations derived from two independent experiments and three replicates per experiment. Asterisks indicate significant (p< 0.05) differences between the groups.

Next step was to analyze the effect of purified PGE_2_ on 24h old *S*. *aureus* biofilms. As shown in Fig 4A, purified PGE_2_ significantly enhanced *S*. *aureus* biofilm formation in a dose-dependent manner. The stimulatory activity of purified PGE_2_ was neutralized by heating treatment of the PGE_2_ solution (Fig 4B).

### *C*. *albicans* PGE_2_-deficient mutant strain do not stimulate growth of *S*. *aureus*

PGE_2_ and its metabolites were abundantly detected in culture supernatants of *C*. *albicans* wt and reference strains, and their amount increased according to the *C*. *albicans* cell density.

**Fig 5:**
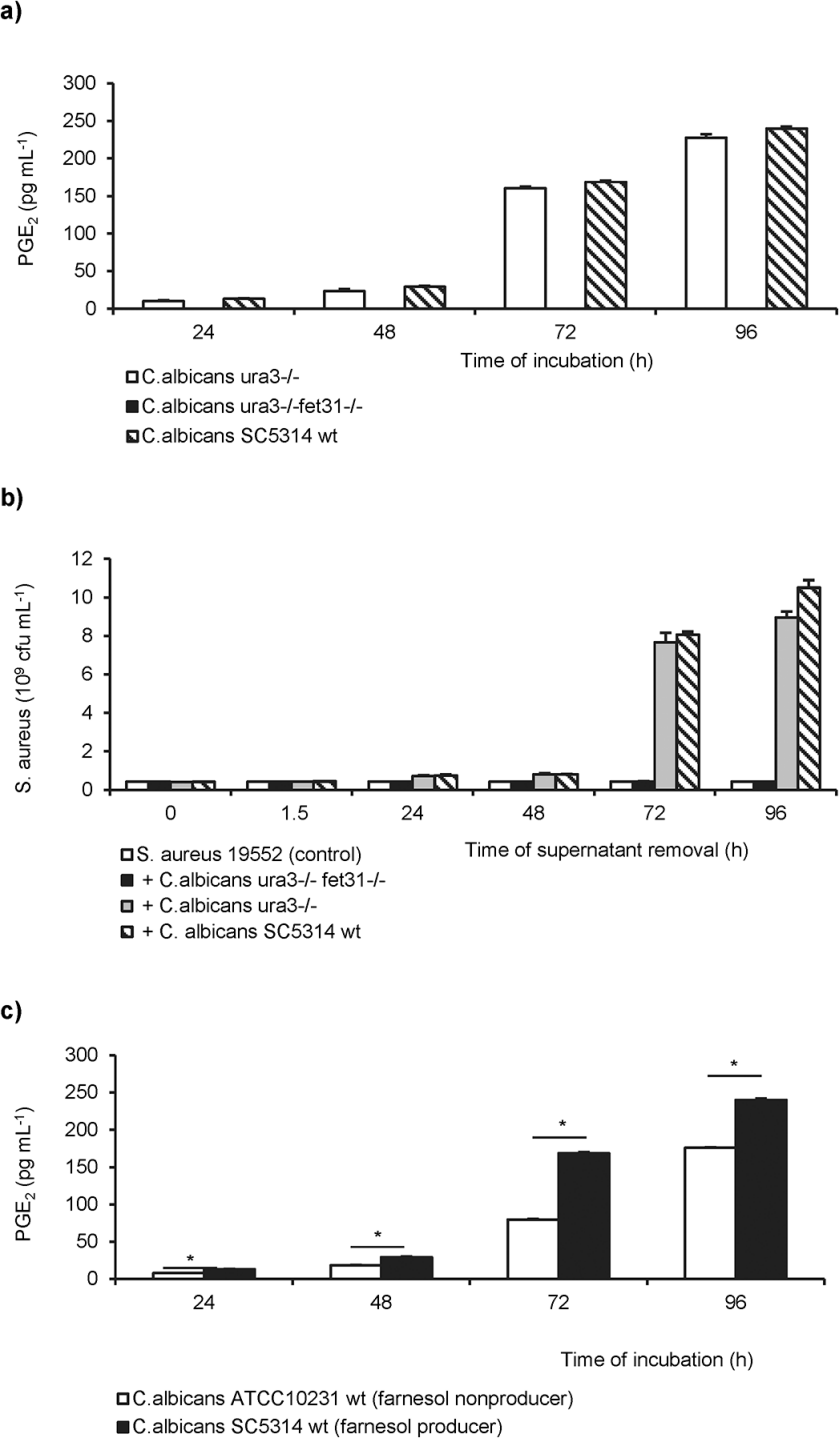
PGE_2_ synthesis in *C*.*albicans* wild type and mutant strains and its effect on *S*. *aureus* biofilms. (**a**) Time-dependent accumulation of PGE_2_ in culture supernatants of the *C*. *albicans* SC5314 wt (dashed), the reference strain *ura3*-/- (open bars), and the null mutant *ura3-/-fet31-/-* (solid bars) measured by EIA. (**b**) Effect of cell-free supernatants derived from *C*. *albicans* SC5314 wt (striped), from the reference (grey bars) and from the null mutant (solid bars) to the growth of established *S*. *aureus* 19552 biofilms (control; open bars) after further 24h of incubation. (**c**) PGE_2_ levels in supernatants of *C*. *albicans* ATCC10231 (farnesol nonproducer; open bars) and SC5314 (farnesol producer; solid bars) were compared. Mean and standard deviations derived from two representative experiments with three replicates per test are demonstrated. Significant differences (*p*<0.001) between the two groups were identified by asterisks.

In contrast, PGE_2_ levels of the *C*. *albicans* null mutant (*ura3-/-fet31-/-*), deficient in the multi-copper oxidase genes *fet31*, were below the detection limit of the assay (Fig 5A and S2 Fig).

The concentration of purified PGE_2_ inducing the growth of *S*. *aureus* were similar to the PGE_2_ levels measured in supernatants of *C*. *albicans* mono-microbial biofilms (Figs 4A and 5A).

To test the hypothesis that PGE_2_ is responsible for the stimulatory effect of *C*. *albicans* on *S*. *aureus* biofilms, the effect of cell-free supernatants derived from PGE_2_ producers (*C*. *albicans* SC5314 and *C*. *albicans ura3-/-)* and from the PGE_2_ nonproducer (*C*. *albicans ura3-/-fet31-/-*) to the growth of *S*. *aureus* was investigated (Fig 5B). Supernatants derived from the PGE_2_-deficient mutant were unable to stimulate the growth of *S*. *aureus*.

Interestingly, the farnesol producer *C*. *albicans* SC5314 secreted significantly higher levels of PGE_2_ than the farnesol-deficient strain ATCC10231 (Fig 5C).

### Treatment of mixed biofilms with indomethacin reduced growth of *S*. *aureus*

**Fig 6:**
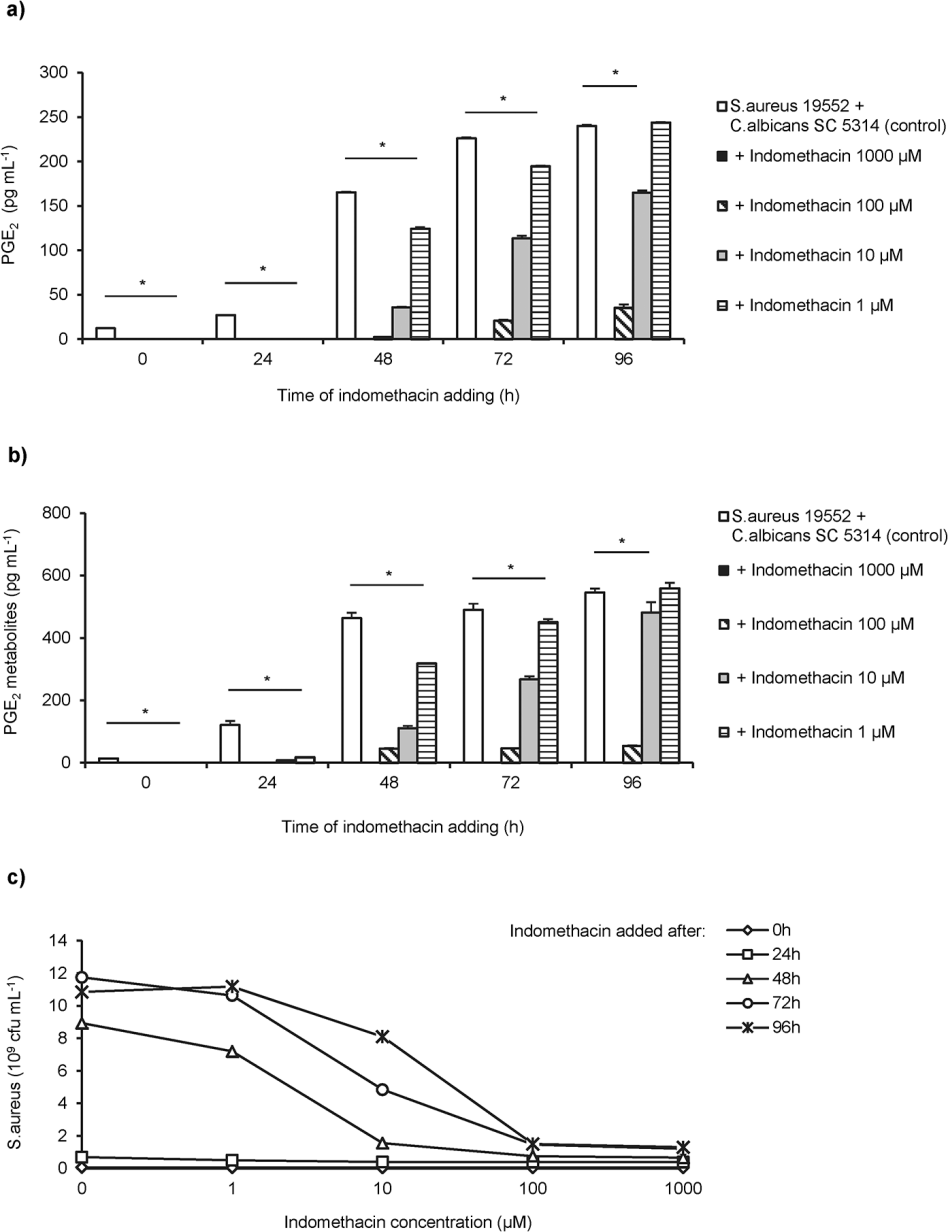
Indomethacin suppresses the stimulatory effect of *C*. *albicans* on *S*. *aureus* biofilms. (**a**) Indomethacin was added to mixed biofilms of *S*. *aureus* 19552 and *C*. *albicans* SC5314 pre-cultured for different periods. PGE_2_ synthesis was measured in supernatants from indomethacin-treated and untreated (control) dual biofilms by monoclonal and (**b**) metabolite EIA. Asterisks indicate significant differences between indomethacin-treated cultures and untreated controls. (**c**) The number of *S*. *aureus* cfu`s was quantified in mixed *S*. *aureus/C*. *albicans* biofilms after addition of graded doses of indomethacin at the indicated time points. Untreated dual species biofilms were used as control. Shown are the results of two representative experiments with three intra-assay replicates.

In addition, we tested whether the nonspecific cyclooxygenase inhibitor indomethacin can influences the stimulatory activity of *C*. *albicans* to *S*. *aureus*. Indomethacin at a concentration between 10 and 1000 pg/mL efficiently blocked the biosynthesis of PGE_2_ (Fig 6A) and its metabolites (Fig 6B) by *C*. *albicans*. In cultures of mixed *S*. *aureus/C*. *albicans* biofilms indomethacin significantly suppressed the growth of *S*. *aureus* in a dose-dependent manner (Fig 6C).

Experiments performed with the *S*. *aureus* small colony variant 31338 revealed similar results.

Neither DMSO nor Indomethacin significantly affected the growth of *S*. *aureus* or *C*. *albicans*. Data are exemplarily represented for *S*. *aureus* 19552 and *C*. *albicans* SC5314 (S3 Fig).

### *S*. *aureus* does not stimulate the PGE_2_ synthesis in *C*. *albicans*

The levels of PGE_2_ and of the PGE_2_ metabolites were not enhanced in dual biofilms of *S*. *aureus* and *C*. *albicans* wild type strains compared with *C*. *albicans* mono-microbial biofilms, indicating that *S*. *aureus* did not stimulate the release of PGE_2_ from *C*. *albicans* isolates (S4 Fig).

## Discussion

In polymicrobial infections, biofilm formation is the predominant mode of life [29–31]. Biofilms are structured microbial communities attached to biotic or abiotic surfaces and embedded in a matrix of exopolymers to withstand the host immune response and exhibit increased resistance to antimicrobial agents [32–35]. It is estimated that 27% of nosocomial *C*. *albicans* bloodstream infections are polymicrobial, with *S*. *aureus* as the third most common organism isolated in conjunction with *C*. *albicans* [36].

*In vitro* studies demonstrated that the formation of *S*. *aureus* biofilms on abiotic materials in the presence of serum requires pre-coating and nutrient supplementation [37]. In order to study the interactions between *C*. *albicans* and *S*. *aureus* in mono-microbial and dual species biofilms, we have optimized an *in vitro* biofilm model of infection. Using this model, we observed increased growth rates of *S*. *aureus* in dual biofilms in the presence of *C*. *albicans*. *In vitro* studies described the formation of *S*. *aureus* microcolonies on the surface of biofilms with *C*. *albicans* serving as the underlying scaffolding [38,39]. The adherence of *S*. *aureus* on fungal hyphae was mediated by the *Candida* agglutinin-like protein 3 (Als3p; [40]). Studies of the protein expression during the growth of dual *C*. *albicans/S*. *aureus* biofilms identified 27 differentially regulated proteins, mostly involved in growth, metabolism, or stress response. Among the down-regulated staphylococcal proteins was the global transcriptional repressor of virulence factors, CodY, suggesting that the enhanced pathogenesis of *S*. *aureus* may not only be due to physical interactions [41,42].

Staib et al. [1] hypothesized the potential existence of *C*. *albicans* end-products as a substratum for a favorable growth of *S*. *aureus*. One of the *C*. *albicans* products continuously excreted in the environment is farnesol a quorum sensing molecule involved in the regulation of the fungal yeast-mycelium dimorphism. At high levels, farnesol has been shown to exhibit antimicrobial activity against various pathogens [12]. Kaneko et al. reported growth inhibition of *S*. *aureus* at concentrations of 40 μg mL^-1^ (equivalent to 180 μM) which is concordant with our results [43]. In this study, subinhibitory concentrations of farnesol (0.5–5 nM) promoted the biofilm formation of *S*. *aureus*, an effect already described for several antimicrobial agents [44–47]. In stationary phase cultures of *C*. *albicans*, farnesol accumulated at levels between 2 and 4 μM [8,48]. In addition, our data showed that *S*. *aureus* biofilm production was propagated by the farnesol-deficient *C*. *albicans* strain ATCC 10231 albeit at a lower level compared to the farnesol producer *C*. *albicans* SC5314, whereas the PGE_2_ null mutant did not enhanced the growth of *S*. *aureus*. Unlike SC5314, ATCC10231 excreted significantly lower levels of PGE_2_ in this experimental setting indicating that the differences in the growth-stimulating effect to *S*. *aureus* resulted from the PGE_2_ levels. In conclusion, it appears that PGE_2_ has a superior role in the induction of *S*. *aureus* biofilm formation as compared to farnesol, which, however, also supports the growth of *S*. *aureus* at low concentrations.

We demonstrated for the first time that *C*.*albicans* PGE_2_ may be one of the suggested end-products presumed by Staib [1]. As shown in this study, the stimulatory effect to the growth of *S*. *aureus* was not observed, when *C*. *albicans* supernatants or medium supplemented with purified PGE_2_ were heat treated suggesting that PGE_2_ is heat-sensitive. This observation is concordant with the results described by Carlson who reported increased mortality rates of mice co-infected with sublethal doses of *C*. *albicans* and *S*. *aureus* [3]. This effect could not be reproduced when either of the agents was heat inactivated.

Endogenous PGE_2_ maintains the integrity of the mucosal barrier in the lungs and in the gastrointestinal tract [49–51]. Mucus accumulation and overexpression of inflammatory response genes are relevant pathogenic features of cystic fibrosis (CF), a genetic disorder caused by mutations in the cystic fibrosis transmembrane conductance regulator gene *cftr* [52]. Recently, Harmon et al. demonstrated a defective peroxisome proliferator-activated receptor (PPARy) function in epithelial cells which was caused by the decreased conversion of PGE_2_ to the PPARy ligand 15-keto-PGE_2_ leading to the accumulation of PGE_2_ [51]. As elevated levels of PGE_2_ have been detected in respiratory tract samples of patients suffering from cystic fibrosis [53,54] and in consideration of the results from this study, it seems to be possible that enhanced PGE_2_ levels may contribute to the early airway colonization due to *S*. *aureus* in patients with CF.

PGE_2_ was demonstrated to induce germ tube formation and to be involved in biofilm formation by *C*. *albicans* [55,56] suggesting *C*. *albicans* PGE_2_ to be a potential virulence factor. Despite induced endocytosis by host cells at the early stage of infection, hyphal mediated active penetration of the host tissue is the major route of invasion [57,58]. Tissue invasion by *C*.*albicans* is associated with cytokine secretion, inducing host immune response mechanisms. Phagocytosis by macrophages and neutrophils represents the first line of defense against *Candida* infections but intracellular killing is not always effective. One of the underlying mechanisms is the inhibition of the phagosome maturation and the nitric oxid (NO) production in macrophages after infection with *C*. *albicans* [59], an effect that may be resulted from candidal PGE_2_ excretion.

PGE_2_ is a critical molecule that regulates the activation, maturation, migration, and cytokine secretion of several immune cells, particular those of the innate immunity. In the context of infection, endogenous PGE_2_ inhibits the cytolytic effector function of natural killer (NK) cells, the activation, migration and production of proteolytic enzymes in granulocytes and limits the phagocytosis and pathogen-killing function of alveolar macrophages (reviewed by [14,18]. Aronoff et al. reported the negative regulatory role of endogenously produced and exogenously added PGE_2_ on FcRy-mediated phagocytosis of bacterial pathogens by alveolar macrophages suggesting that PGE_2_ derived from *C*. *albicans* may impair the local host innate immunity [60]. This suggestion is underlined by the investigations of Roux et al. who had shown that airway colonization with *C*. *albicans* inhibited phagocytosis of *S*. *aureus* and *P*. *aeruginosa* and enhanced the prevalence of bacterial pneumonia, which was reduced by antifungal treatment [61,62].

Mice inoculated with either *S*. *aureus* or *C*. *albicans* survived infection, whereas combined infection with both pathogens enhanced the mortality rate to 40–100% [3,4]. Reversely, enhanced microbial clearance and survival was demonstrated in studies with COX-2-deficient mice [63–65]. This is in line with our observation that a mutant strain of *C*. *albicans* deficient in PGE_2_ production did not promote the growth of *S*. *aureus*. Furthermore, in this study the non-selective cyclooxygenase (COX) inhibitor indomethacin that blocked PGE_2_ biosynthesis by *C*. *albicans* also reduced the growth of *S*. *aureus* in dual biofilms to a level observed in mono-microbial *S*. *aureus* biofilms.

Thus, treatment with indomethacin or with antifungal agents may exhibit several positive effects in patients with dual *S*. *aureus*/*C*. *albicans* infections [51,60–62].

Finally, in this study *S*. *aureus* did not enhanced the biofilm thickness of *C*. *albicans* and its PGE_2_ synthesis in dual biofilms compared with mono-microbial *C*. *albicans* biofilms, although bacterial peptidoglycan-derived molecules have been shown to promote *C*. *albicans* hyphal growth [66,67]. Thus, in mixed biofilms the impact of *S*. *aureus* to *C*. *albicans* remained unclear.

## Conclusion

Our findings indicate that PGE_2_ is the key molecule stimulating the growth and biofilm formation of *S*. *aureus* in dual *S*. *aureus/C*. *albicans* biofilms, although subinhibitory farnesol concentrations may also support this effect. Candidal PGE_2_ may exhibit a dual effect in *S*. *aureus/C*. *albicans* polymicrobial biofilms, first, by promoting fungal hyphal formation and second, by providing a proper substratum for the proliferation of *S*. *aureus*. Further characterization of the intricate interaction between these pathogens is warranted, as it may aid in the design of further therapeutic strategies against polymicrobial biolfilm infections.

## Supporting Information

S1 Fig Effect of farnesol on the growth of *S*. *aureus*. (TIF)

S2 Fig PGE_2_ metabolite synthesis in *C*. *albicans* wt strains. (TIF)

S3 Fig Indomethacin and DMSO does not affect the growth of (a) *S*. *aureus* or (b) *C*. *albicans*. (TIF)

S4 Fig *S*. *aureus* does not stimulate the PGE_2_ synthesis in *C*. *albicans*. (TIF)
