# Supplementary figures and images for: Prostaglandin E2 from Candida albicans Stimulates the Growth of Staphylococcus aureus in Mixed Biofilms
Source: PLoS One. 2015 Aug 11;10(8):e0135404. doi: 10.1371/journal.pone.0135404 (PMC4532413; doi:10.1371/journal.pone.0135404)

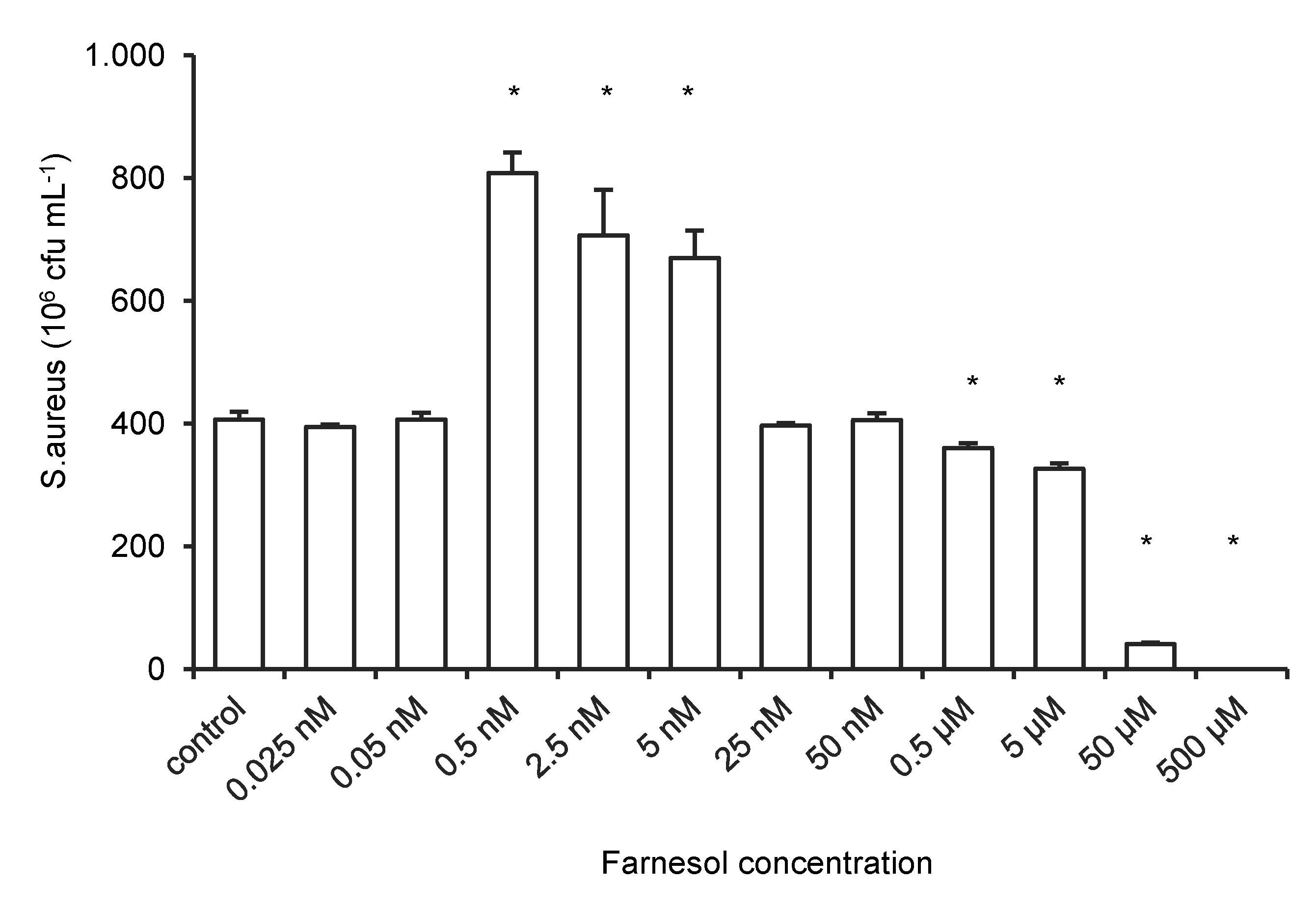

Supplement: S1 Fig — (TIF) [file pone.0135404.s001.tif]

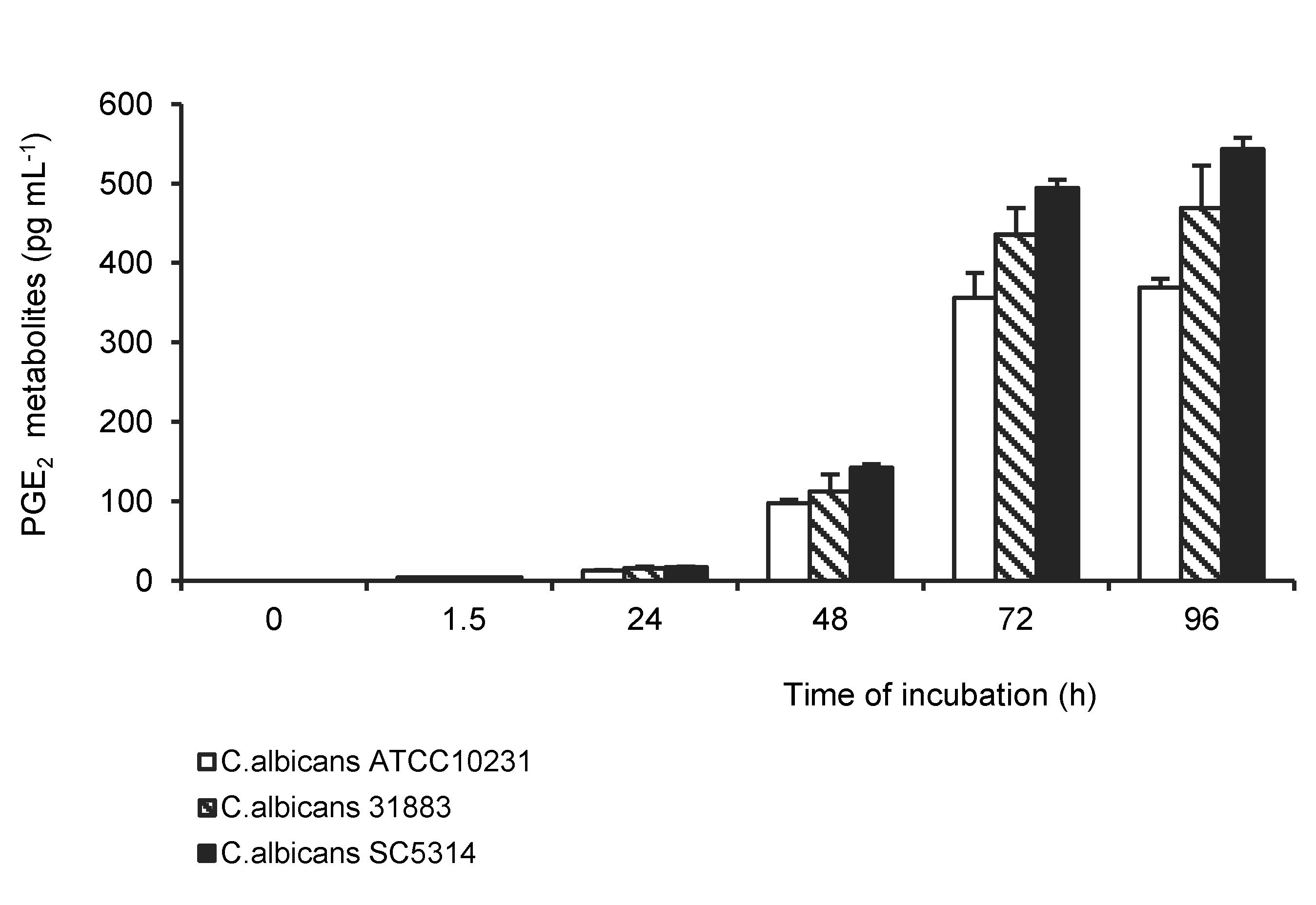

Supplement: S2 Fig — (TIF) [file pone.0135404.s002.tif]

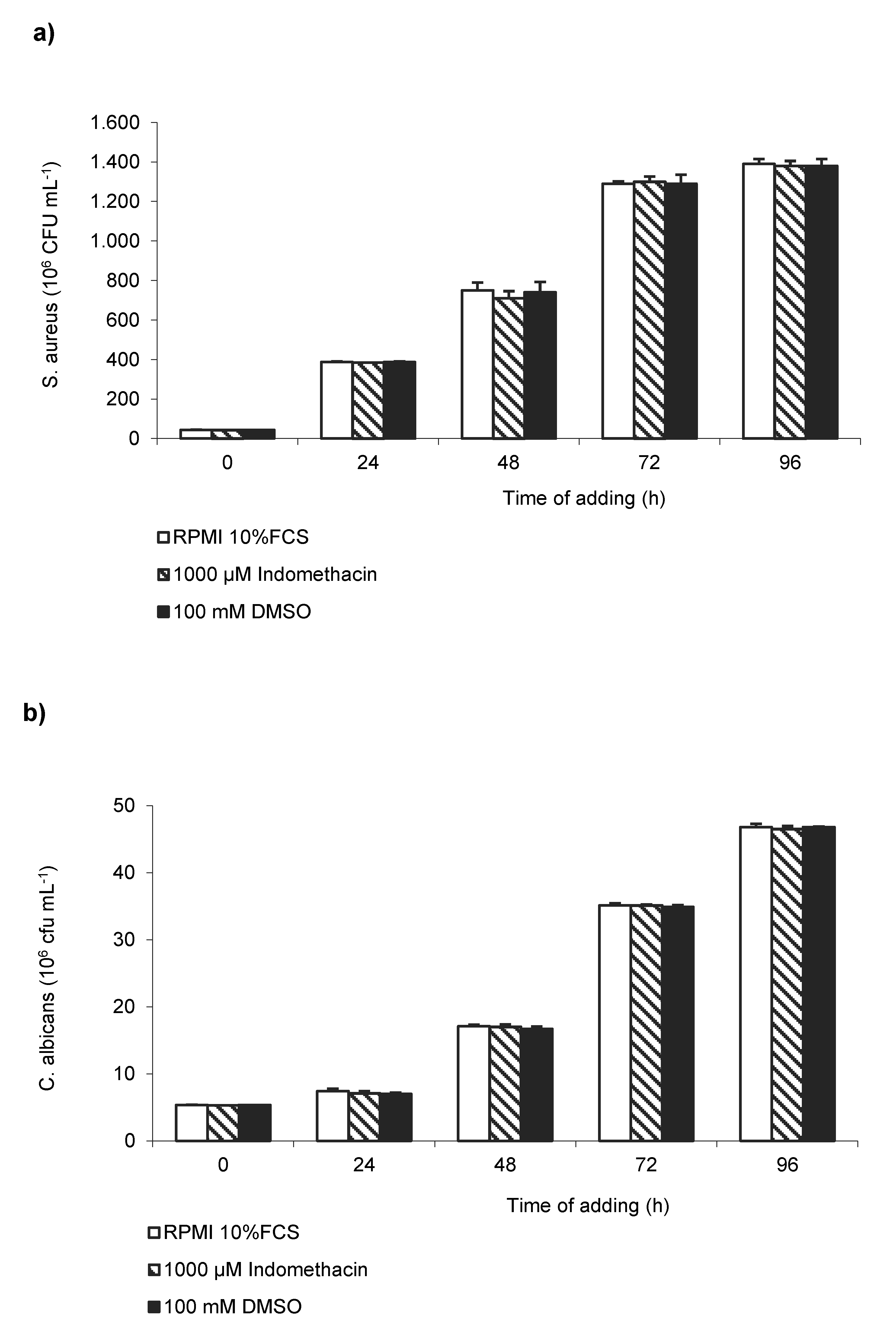

Supplement: S3 Fig — (TIF) [file pone.0135404.s003.tif]

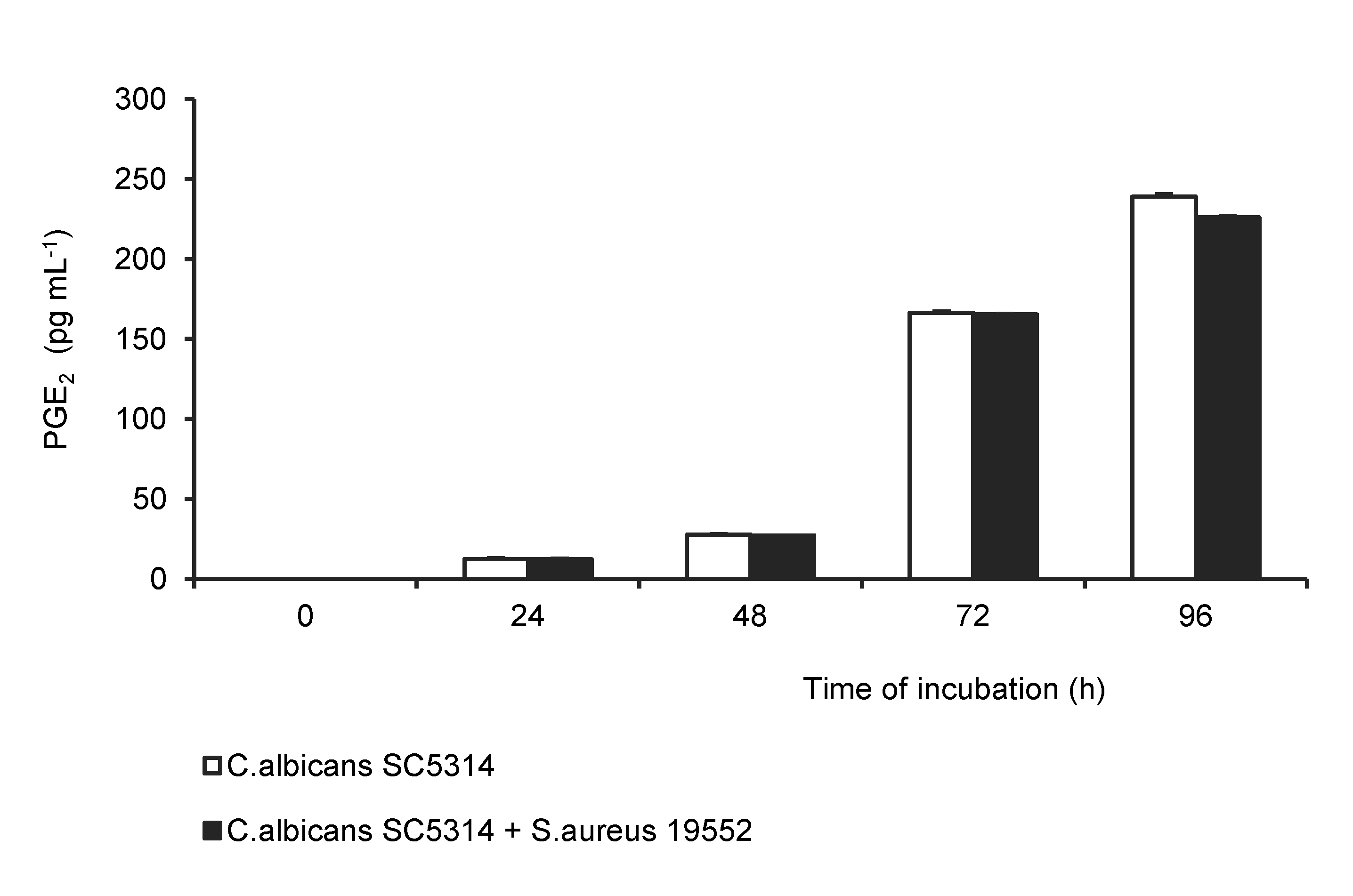

Supplement: S4 Fig — (TIF) [file pone.0135404.s004.tif]
